# Diagnostic value of optical coherence tomography for intracranial pressure in idiopathic intracranial hypertension

**DOI:** 10.1186/1129-2377-14-S1-P166

**Published:** 2013-02-21

**Authors:** M Skau, H Yri, T Gerds, R Jensen

**Affiliations:** 1 Danish Headache Centre, Department of Neurology, Denmark; 2 Department of Biostatistics Copenhagen, University of Copenhagen, Denmark, Denmark

## Introduction

Idiopathic intracranial hypertension (IIH) primarily affects young obese women. It can cause chronic headache and permanent visual loss due to papilloedema and secondary optic atrophy.

## Purpose

To evaluate the diagnostic value of optical coherence tomography (OCT) as a marker of cerebrospinal fluid (CSF) opening pressure in patients with IIH.

## Methods

We investigated CSF opening pressure, peripapillary retinal nerve fibre layer thickness (RNFLT), total retinal thickness (RT), and headache symptoms in 20 patients newly diagnosed with IIH, 21 patients with long-term IIH, and 20 healthy controls. The diagnostic ability of OCT as a marker of increased ICP (> 25 cmH2O) was explored by multiple regression analyses and receiver operating characteristic (ROC) curves. As a new diagnostic tool, we developed an OCT elevation diagram.

## Results

OCT elevation diagrams showed that in 60% of patients newly diagnosed with IIH and in 10% of patients with long-term IIH, 50% or more of the OCT scans were above normal. The percentage of abnormal OCT scans was significantly associated with increased opening pressure (p<0.0001). By including OCT in the multiple regression model, the estimated areas under the ROC curves increased from 77.1 to 86.9. Headache was a presenting symptom in 71% of patients. In 59% it was the only early indication of IIH. Persistence of headache was seen in 70% of patients with long-term IIH, of whom 50% reported constant headache.

## Conclusion

Increased peripapillary retinal thickness measured by OCT is closely associated with increased ICP in newly diagnosed IIH patients. Thus OCT could be a valuable diagnostic test in the very subjective assessment of papilloedema in patients suspected of having IIH. However, in patients with long-term IIH, OCT is of limited value in predicting ICP. Headache is an important early symptom of IIH, which despite treatment, persists and disables the vast majority of patients.

